# Should Denosumab or Radical Surgery Be Recommended for a Spinal Giant Cell Tumour in a Fertile Female?

**DOI:** 10.7759/cureus.11526

**Published:** 2020-11-17

**Authors:** Harshadkumar Dhirajlal Rajgor, Steven James, Rajesh Botchu, Melvin Grainger, Marcin Czyz

**Affiliations:** 1 Orthopaedics, Royal Orthopaedic Hospital, Birmingham, GBR; 2 Radiology, Royal Orthopaedic Hospital, Birmingham, GBR; 3 Neurosurgery, University Hospitals Birmingham, Birmingham, GBR

**Keywords:** giant cell tumour of bone, spinal tumor

## Abstract

Giant cell tumour (GCT) of the spine is a benign aggressive tumour with high recurrence rates. Patients can be asymptomatic due to the slow growth rate and present with localized pain or neurological dysfunction. Current management strategies include intralesional curettage, total en-bloc resection (TER) and denosumab therapy. Treatment strategies can be particularly challenging in women of childbearing age who wish to conceive, as the risks of tumour recurrence need to be balanced against the fetal complications associated with adjuvant denosumab therapy. This case report discusses the management options and controversies for women of childbearing age with GCT of the thoracic spine. Clinicians need to be aware of the complications associated with TER and denosumab treatment when managing GCTs of the spine in young females.

## Introduction

Tumours of the osseous spine account for 4.6% of bone tumour cases. Spinal giant cell tumours (SGCTs) account for 7%-10% of all spinal tumours and most commonly occur in the sacrum. SGCTs represent less than 5% of all giant cell tumours (GCTs) [1]. GCTs are more common in females at a median age of 35 years. GCTs tend to be benign tumours that can be locally aggressive, with a potential to metastasize in 3% of cases [2].

GCTs are well-vascularized and contain collagenous fibrous tissue. Macrophages, areas of necrosis and haemosiderin deposits are present macroscopically in large GCTs. Ossification at the periphery of the lesion is commonly identified radiologically. Aneurysmal bone cysts also occur in 10%-14% of GCTs of bone [3]. GCTs microscopically contain osteoclast precursor cells and neoplastic stromal cells. The over-expression of the RANK ligand by neoplastic stromal cells can be targeted by denosumab [4].

Patients with SGCT can be asymptomatic and present with localized pain or neurological symptoms. Early diagnosis can be difficult due to the insidious onset and slow growth rate [5]. Neurological compromise can occur due to nerve or canal involvement along with cord compression due to soft tissue extension of the tumour. This can lead to patients presenting with sensory disturbances or motor symptoms. Urinary incontinence can also occur if compression of the cauda equina is present [6]. MRI and CT are vital for early diagnosis and surgical planning. The differential diagnosis for SGCT includes chordoma, aneurysmal bone cysts, osteosarcoma and metastatic lesions [7].

This case report discusses the management options, complications and controversies for women of childbearing age with GCT of the spine, with a particular focus on total en-bloc resection and the use of denosumab.

## Case presentation

A 24-year-old school teacher with a history of mild asthma presented to her local emergency department with a nine-month history of right-sided chest pain in the T7 nerve distribution. She also experienced severe, constant mid-thoracic spinal pain, which was exacerbated by elevation of her right arm. The pain was causing a significant restriction to her daily activities, leading to a four-week absence from work. Examination findings demonstrated tenderness on palpation of her mid-thoracic spine. No neurological deficit or upper motor neurone signs were identified in the upper or lower limbs.

Diagnostic imaging

**Figure 1:**
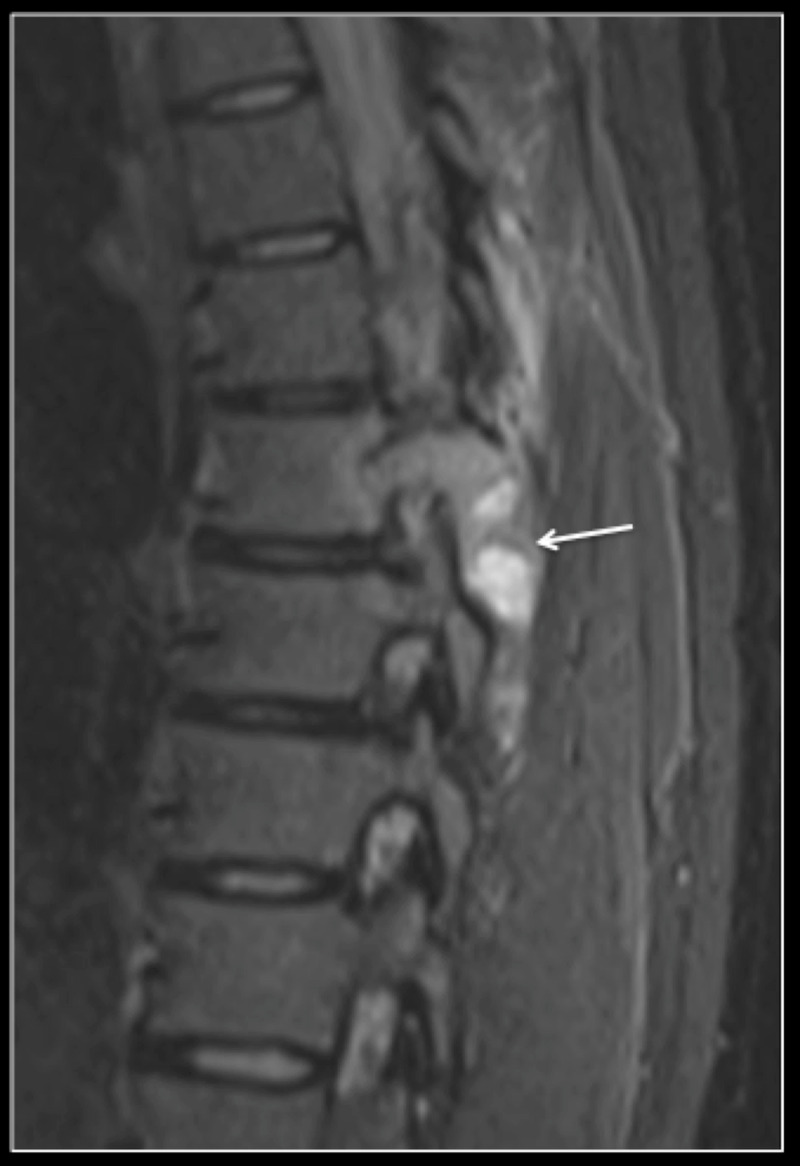
Sagittal STIR MRI of the thoracic spine showing a tumour (arrow) within the right pedicle and lamina with marked perilesional soft tissue oedema STIR: short-TI inversion recovery; MRI: magnetic resonance imaging

Magnetic resonance imaging (MRI) whole spine demonstrated a lesion involving the right inferior articular process of T7 extending into the right pedicle, the posterior portion of the vertebral body, the right hemi-lamina and the head of the seventh rib. Marked perilesional oedema was evident (Figure 1).

**Figure 2:**
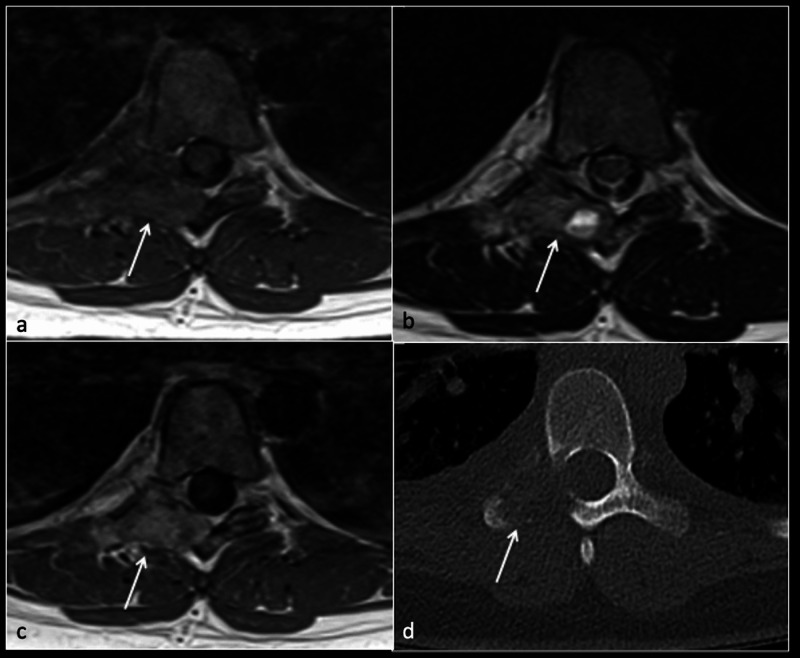
Axial T1 (a), T2 (b), T1 post-contrast (c), and CT (d) showing tumour (arrow) in the right pedicle and lamina with fluid level CT: computed tomography

The lesion had a mixed solid and cystic appearance with multiple fluid-filled levels, suggestive of an SGCT. The surrounding bone appeared expanded, extending into the right lateral aspect of the canal. No compression of the neural structures was present (Figure 2).

A staging computed tomography (CT) scan showed no evidence of distant metastasis. The CT scan of the thoracic spine showed a destructive lesion of T7 involving the right lamina and transverse process (Figure 2). Subsequent CT-guided biopsy via a midline approach, to aid surgical resection, confirmed an SGCT.

A spinal oncology multi-disciplinary team (MDT) involving spinal tumour surgeons, radiologists and specialist nurses discussed surgical, medical and conservative management options. Medical management with denosumab was discussed, including the possible risk of neurological deterioration caused by progressive tumour growth leading to compression of the spinal cord or nerve roots. Information was provided highlighting the effects of denosumab during pregnancy, as she was looking to conceive in the near future. Two surgical options were discussed: total en bloc resection (TER) of tumour and intralesional curettage. The patient underwent a TER of SGCT of T7 along with posterior instrumented fusion at a tertiary spinal unit.

Pre-operative embolization was carried out 24 hours prior to surgery to reduce intra-operative blood loss. Diagnostic angiography showed tumour blush in the right pedicle. The artery feeding the tumour was coil embolized with a 4 mm x 7 cm micro nester coil. There was no major blood supply to the cord from the right intercostal arteries one level above and below, and they were coil embolized beyond branches feeding the tumour blush. No post-embolization complications occurred.

En-bloc resection of SGCT of T7 along with posterior instrumented fusion was successfully performed. A midline posterior approach was used with the patient placed prone on a Jackson table under general anaesthetic. The appropriate level was marked using X-ray guidance followed by a vertical incision. The initial biopsy track was excised with margins of the surrounding tissue. Fine soft tissue dissection followed with adequate exposure of T5-10 on the left, T5-6 and T9-10 on the right. Instrumentation of T5, T6, T9 and T10 with pedicular screws followed. The seventh and eighth ribs were exposed on the right 2 cm lateral to the transverse process (TP). The eighth rib was removed down to the TP with the seventh rib being cut 1 cm lateral to the TP. Parietal pleura was identified and protected throughout the course of the procedure. The eighth thoracic nerve root was identified along with the eighth segmental artery, which was ligated. T6 and T8 laminectomies were performed with a left hemilaminectomy of T7. T6/7 and T8/9 facetectomies on the right occurred. The dura was exposed, and the tumour was visible. The pedicle was undercut with the Sonopet Ultrasonic (Stryker Corporation, Kalamazoo, Michigan) at its base. The T6/7 disc was cut with an osteotome. The specimen was flipped over the dura with thoracic nerve root 7 ligated. A small dural tear occurred at this point, which was securely repaired. Extensive epidural vein bleeding was evident, which was controlled by bipolar cauterization and packing. Titanium 6.35 kyphotic rods were fit between T5 and T10 bilaterally. The femoral head graft was secured into the cavity after partial vertebrectomy.

The patient was mobilized on the second postoperative day. Unfortunately, on day three, she developed progressive right-sided chest pain and shortness of breath A plain film chest radiograph and subsequent CT scan revealed pneumothorax, which required a chest drain. There was no evidence of an infection or active bleeding, vital parameters improved immediately and the patient was discharged home eight days later.

She was placed on a physiotherapy regime and made good functional progress. However, her right-sided chest pain persisted, which was attributed to intercostal neuralgia. She was subsequently referred for an intercostal nerve block achieving nearly complete and permanent resolution of pain. Two-years post-operatively, she was pain-free and there was no evidence of recurrence.

## Discussion

Surgical management of SGCTs can be potentially curative. SGCT can be treated with intralesional curettage, piecemeal spondylectomy or by performing a TER. Ouyang et al. demonstrated the recurrence rate of SGCTs to be as high as 80% in those undergoing intralesional curettage, 8.8% in those undergoing piecemeal spondylectomy and 0% in those undergoing TER [8]. TER provides the benefit of wider surgical margins and macroscopic clearance. TER is a technically demanding procedure which carries a higher complication rate compared to intralesional curettage. Literature observes that complications occur in 46.2% of patients following en-bloc resection with an associated rate of mortality of 4.6% [9]. Major complications of TER are reported to be 8%. These include pneumothorax, paraplegia, aortic injury, major haemorrhage and deep wound infection [10].

Denosumab, a monoclonal antibody, has shown to be an effective modality of treatment in patients with a significant risk of postoperative morbidity or unresectable disease due to tumour size. Neo-adjuvant therapy with denosumab can downstage disease for those requiring extensive surgery. Chawla et al. have also reported that denosumab therapy inhibited disease progression in patients with surgically unsalvageable GCT in 163/169 (96%) of cases [11]. Tumour response is evident radiologically at 24 weeks following denosumab treatment in 68.5% of cases [12]. It is also used for adjuvant treatment following intralesional curettage [13]. Martin-Broto et al. reported a reduction in limb pain following the use of denosumab in a cohort of 170 patients at 30 days with GCT [14].

SGCTs are the most common type of pregnancy-related primary spinal tumours (PRST) followed by hemangioma and schwannoma. The management of spinal tumours during pregnancy can be challenging for the surgical team [15]. Currently, no consensus on the duration of treatment of denosumab use in SGCTs pre or postoperatively has been reached [16]. The Food and Drug Administration (FDA) has recommended the use of contraception for at least five months after the last dose of denosumab [17]. Boyce et al. observed that in-utero exposure to denosumab in cynomolgus monkeys led to a decrease in long bone length, long bone fractures due to a reduction in cortical thickness and dental dysplasia secondary to growth impairment of the mandible. In-utero exposure to denosumab is also associated with an increased risk of stillbirth and growth abnormalities. Other adverse effects include jaw osteonecrosis, hypophosphataemia and hypocalcaemia [18].

## Conclusions

We reported a rare case of SGCT of the thoracic spine. When discussing surgical treatment, consideration is given to adjuvant denosumab treatment regularly. Women of childbearing age should be proactively counselled regarding the associated risks of denosumab treatment during pregnancy or if trying to conceive. These are important questions that may need to be answered from a patients’ perspective and balanced against the risks of tumour progression.
